# Prevalence of Papillary Thyroid Carcinoma is Significantly Higher in Graves Disease with Synchronous Thyroid Nodules*

**DOI:** 10.5146/tjpath.2024.13650

**Published:** 2024-09-02

**Authors:** Mehmet Kefeli, Hasan Gucer, Elif Tutku Durmuş, Aysegul Atmaca, Ramis Colak, Ozgur Mete

**Affiliations:** Department of Pathology, Ondokuz Mayis University, Samsun, Türkiye; Department of Pathology, Recep Tayyip Erdogan University, Rize, Türkiye; Department of Endocrinology and Metabolism, Ondokuz Mayis University, Samsun, Türkiye; Endocrine Oncology Site Group, The Princess Margaret Cancer Centre, Toronto, On, Canada; Department of Laboratory Medicine and Pathobiology, University of Toronto, Toronto, On, Canada; Department of Pathology, University Health Network, Toronto, On, Canada

**Keywords:** Graves disease, Papillary thyroid carcinoma, Thyroid carcinoma, BRAF-like, RAS-like

## Abstract

***Objective: *** The association between autoimmunity-related tissue injury and thyroid cancer development remains an area of interest. Evidence suggests that patients with Graves disease (GD) may have an elevated risk for differentiated thyroid cancer. Multicenter studies are needed to gain insight into the correlates of papillary thyroid carcinoma (PTC) identified in this particular group of patients. This study aimed to investigate the prevalence of PTC and synchronous thyroid nodules in thyroidectomy specimens from GD patients in an endemic goiter region.

***Material and Methods:*** A retrospective review of institutional pathology records at two tertiary care centers identified 237 surgically treated patients with GD. Patients were categorized as having nodular Graves disease (N-GD) if synchronous nodular thyroid was identified by ultrasonography, while those without synchronous thyroid nodules were categorized as non-nodular or simple Graves disease (S-GD). The prevalence of PTC, histopathological correlates, and demographic characteristics were recorded and compared between groups N-GD and S-GD.

***Results: *** One hundred thirty-one and 106 patients were assigned to N-GD and S-GD, respectively. The mean age was significantly higher in N-GD (mean 45.52 years) compared to S-GD (mean 35.18 years) (p<0.001). The overall frequency of PTC was 36.3% (86/237) in the entire cohort. PTC was identified in 48.1% (63/131) of N-GD and 21.7% (23/106) of S-GD (p<0.001). Subcentimeter tumors constituted the majority of cases in both groups (76.2% in N-GD and 82.6% in S-GD) (p>0.05). The group of S-GD was enriched in BRAF-like PTCs, whereas N-GD had equal distribution for RAS- and BRAF-like tumors.

***Conclusion:*** This study underscores that the majority of PTCs encountered in GD were enriched in low-risk subcentimeter PTCs with a prevalence that varies depending on the presence of underlying nodular thyroid tissue.

## INTRODUCTION

Graves disease (GD), also known as diffuse hyperplasia, is an autoimmune endocrine disease characterized by excessive production of thyroid hormones and diffuse enlargement of the thyroid gland (1). The estimated prevalence of GD in the US population is approximately 0.4%-1% (2,3). The pathogenesis of GD involves the production of stimulating TSHR autoantibodies and the presence of immune cells reactive to the TSHR antigen. Excessive autoantibody production leads to TSHR activation, resulting in increased thyroid hormone synthesis, follicular cell proliferation, and diffuse gland enlargement. This process is accompanied by a complex interaction between T lymphocytes and thyroid follicular cells, which leads to tissue injury (4,5).

The role of autoimmunity-related tissue injury and thyroid cancer development is an area of interest. Studies suggested that patients with GD may have an increased risk of differentiated thyroid cancer, with incidence rates ranging from 2-33.7% in case series (6–13). While thyroid glands are usually diffusely enlarged in GD, nodules can also be present and detected through palpation or imaging studies (14–17). Some series have reported that the follicular cell-derived thyroid carcinoma incidence is more frequently in thyroid nodular lesions when compared to incidental follicular cell-derived thyroid carcinoma in patients with GD without nodules (6–8,18–20).

Multicenter studies are much needed to gain insights into the correlates of papillary thyroid carcinoma (PTC) identified in this particular group of patients. For this reason, the current study investigated the prevalence of PTC and synchronous thyroid nodules in thyroidectomy specimens of GD patients from an endemic goiter region.

## MATERIAL and METHODS

A retrospective review of institutional pathology records at two tertiary care centers identified 237 surgically treated patients with GD who underwent thyroidectomy between 2010 and 2019 at Ondokuz Mayis University (n = 139) and Recep Tayyip Erdogan University (n = 98). Institutional REB (research ethics board) approvals were obtained. In the process of data collection, the following information was recorded: demographic data of the patients, medical histories and treatments, results of neck ultrasonography (US) (presence or absence of thyroid nodules), thyroid scintigraphy/radioactive iodine uptake, measurements of thyroid function [levels of free triiodothyronine (fT3), free thyroxine (fT4), and thyroid-stimulating hormone (TSH)], thyroid autoantibodies (anti-thyroglobulin, antithyroid peroxidase, and thyroid-stimulating hormone receptor antibodies), indications and notes for all operations, and histopathological results. The diagnoses of GD were made based on relevant medical histories and signs of hyperthyroidism, laboratory findings of reduced TSH with elevations of fT3/fT4, identification of thyroid autoantibodies in serum, and increased uptake of radioactive iodine or diffuse uptake according to thyroid scintigraphy at the time of admission. Additional assessment also included the search for ophthalmopathy, and colored-flow or power Doppler US was performed as necessary to confirm the diagnosis. Observations, such as hypoechogenicity and diffuse thyroid enlargement, were noted on US and were considered along with observations from power Doppler US (21). None of the participating patients had undergone radioiodine therapy or external irradiation of the neck. Two patient groups were included in the study, with participants divided based on the US findings. Patients were grouped as having N-GD when a coexistent nodular thyroid was identified by thyroid ultrasonography, and those without synchronous thyroid nodules were grouped as simple GD (S-GD). The prevalence of PTC and its histopathological and demographic correlations were compared between the two groups. PTCs with exclusive follicular architecture were assigned to RAS-like PTCs whereas tumors with classic architecture and/or special cytomorphology (e.g., tall cell) were assigned to BRAF-like PTCs. Tumors with oncocytic and clear cell change were also subtyped as BRAF-like or RAS-like based on their cytomorphological features (22).

For data analysis, IBM SPSS Statistics 21.0 (IBM Corp., Armonk, NY, USA) was used. Continuous variables are presented as mean±standard deviation (SD) and median (range); categorical variables are expressed as percentages. The Mann-Whitney U test was used to evaluate numerical data such as age and tumor size and the chi-square test was used for all other comparisons. Statistical significance was defined as p<0.05.

## RESULTS

Out of the 237 patients enrolled in this study, 160 (67.5%) were female and 77 (32.5%) were male. The mean age of the patients was 40.89±13.41 years, with a of 15 to 76 years. Thyroid nodules were detected in 131 (55.3%) patients, while no nodules were detected in 106 (44.7%) patients. Within the N-GD group, 95 (72.5%) patients were female, 36 (27.5%) were male, with a mean age of 45.52±13.37 years. In S-GD, 65 (61.3%) were female, and 41 (38.7%) were male, with a mean age was 35.18±11.09 years. The mean age was significantly higher in N-GD (p<0.001).

**Figure 1:**
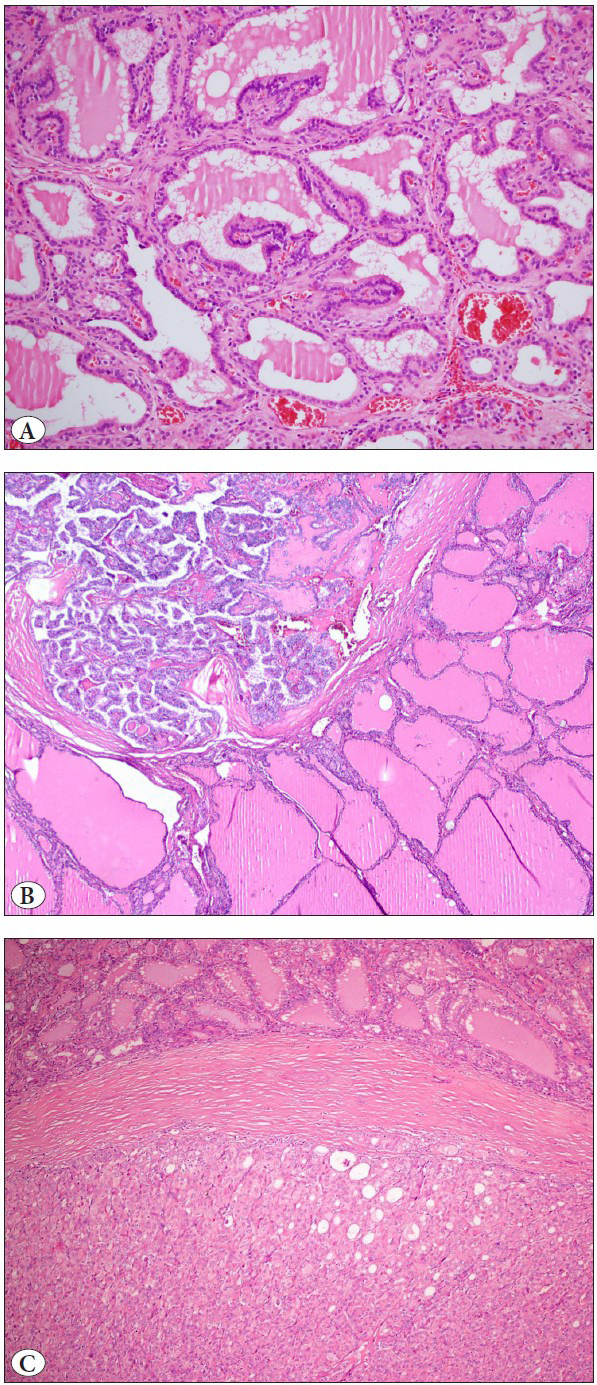
Graves disease is characterized by diffuse hyperplasia **(A).** BRAF- and RAS-like papillary thyroid carcinomas identified in Graves disease **(B,C).**

PTC was detected in 86 of 237 patients (36.3%). The PTC rates were 48.1% (63/131) and 21.7% (23/106) in N-GD and S-GD, respectively. The frequency of PTC was significantly higher in N-GD (Figure 1) (p<0.001). Tumor diameter ranged from 0.01 cm to 5.5 cm. Subcentimeter tumors (formerly known as papillary microcarcinomas) accounted for approximately 76.2% (48/63) of cases in N-GD and 82.6% (19/23) of cases in S-GD (p>0.05). Both N-GD and S-GD showed similar patterns in terms of PTC variants (invasive encapsulated follicular variant) or subtypes (classic, oncocytic, tall cell, and clear cell), bilaterality, multifocality, tumor localization, vascular invasion, lymphatic invasion, perineural invasion, parenchymal invasion, microscopic extrathyroidal extension, and cervical lymph node metastasis (p>0.05). Although not statistically significant, S-GD was enriched in BRAF-like PTCs, whereas N-GD had an equal distribution with respect to RAS and BRAF-like PTCs. The clinicopathological and histopathological features of the patients are summarized in Table 1.

**Table 1:** Clinical and histopathological characteristics in Graves disease with and without thyroid nodule.

	**Nodular GD (n=131)**	**Simple GD (n=106)**	**p value**
Age (years) (mean±SD)	45.52±13.37	35.18±11.09	**<0.001**
Gender, n (%)			
Female	95 (59.4)	65 (40.6)	0.067
Male	36 (46.8)	41 (53.2)	
PTC, n (%)	63 (73.3)	23 (26.7)	**<0.001**
Tumor size (cm) (mean±SD)	0.76 ± 0.90	0.73 ± 0.99	0.384
≤1 cm	48 (71.6)	19 (28.4)	0.733
>1 cm	15 (78.9)	4 (21.1)	
Subtypes/variants, n (%)			
Follicular variant	20 (74.1)	7 (25.9)	0.908
Classic subtype	27 (67.5)	13 (32.5)	0.171
Oncocytic follicular variant	6 (85.7)	1 (14.3)	0.669
Tall cell subtype	7 (87.5)	1 (12.5)	0.676
Clear cell subtype	3 (100)	0 (0)	0.561
Bilaterality, n (%)	16 (84.2)	3 (15.8)	0.353
Multifocality, n (%)	24 (70.6)	10 (29.4)	0.839
Tumor localization, n (%)			
Right	42 (77.8)	12 (22.2)	0.328
Left	33 (73.3)	12 (26.7)	0.986
Isthmus	4 (66.7)	2 (33.3)	0.656
Encapsulated, n (%)			
Total	16 (84.2)	3 (15.8)	0.353
Partial	8 (88.9)	1 (11.1)	0.434
Vascular invasion, n (%)	0 (0)	1 (100)	0.267
Lymphatic invasion, n (%)	8 (80)	2 (20)	0.999
Perineural invasion, n (%)	0 (0)	2 (100)	0.069
Parenchymal invasion, n (%)	29 (65.9)	15 (34.1)	0.183
Extrathyroidal extension, n (%)	2 (100)	0 (0)	0.999
Surgical margin intact, n (%)	4 (66.7)	2 (33.3)	0.656
Cervical lymph node metastasis, n (%)	7 (87.5)	1 (12.5)	0.668
Cervical lymph node dissection, n (%)	14 (60.9)	9 (39.1)	0.211

**GD:** Graves disease, **PTC:** Papillary thyroid carcinoma.

## DISCUSSION

The association between follicular cell-derived thyroid carcinoma and GD has been an area of interest in endocrine oncology. Several pathogenetic hypotheses have been considered. Thyroid-stimulating hormone (TSH) and potentially multiple other factors may be responsible for the development of thyroid cancer in GD (23).

TSHR-stimulating antibodies with strong agonistic activity to TSHR are produced in GD, and the resulting antibody-mediated TSH stimulation causes the production of thyroid hormones. These antibodies also play a role in the proliferation and activity of thyroid follicular cells by inducing a TSH-like effect. Morshed et al. demonstrated that TSH stimulated by autoantibodies activated cAMP, CREB, AKT, and PKC and induced FRTL-5 rat thyroid cell proliferation in cell cultures (24). These findings suggest that multiple mitogenic factors are likely involved in thyroid follicular cell activation and proliferation, and these factors may be responsible for promoting tumor growth, inducing invasion, and stimulating angiogenesis in partnership with other growth factors (24–28). In some instances, the extensive sampling of tumors may reveal subtle microscopic foci of PTCs (29).

The characteristic presentation of GD typically involves diffuse enlargement of the thyroid gland, although nodular presentation is also not uncommon. The frequency of thyroid nodules ranged from 15 to 50% of GD, depending on the detection methods used to identify the presence of synchronous nodular thyroid gland (palpation, ultrasonography, or scintigraphy) (6,8,14–17,20,30). Chen et al. observed a 1.37-fold higher incidence of thyroid cancer in a GD cohort than in a control cohort (10). Some thyroid carcinomas have been incidentally detected in GD patients undergoing thyroidectomy, while others have been identified in the pre-operative workup of N-GD. In some series, PTC incidence ranged from 6.8% (8/117) to 17% (8/48) in GD (31,32), whereas in other series a much higher incidence (>30%) was reported (6,33,34). Similarly, we noted a rate of 36.3% (86/237) in our cohort. The variability in malignancy rates across these series may be attributed to geographic variation, variations in the extent of gross specimen sampling and pathologist experience (29,35).

Former studies have indicated that the incidence of PTC is much higher (ranging from 17.1% to 69%) in patients with underlying thyroid nodules (6,18,19) compared to those without nodules. This observation has also been supported in a meta-analysis that showed thyroid carcinoma occurrence in almost five times more frequently in GD with synchronous thyroid nodules (8). Consistent with the former data, our series also showed a higher frequency of PTCs in N-GD (48.1%) compared to S-GD (21.7%).

The biological behavior difference of carcinoma observed in GD and euthyroid patients has also been area of debate in the literature. Some studies reported thyroid carcinoma in GD can be associated with aggressive biological behavior and a worse clinical outcome when compared to euthyroid patients with differentiated thyroid carcinoma (11,26,36–39). However, these results have not been widely supported in all former series (40–42). While the current study design precludes us to assess the biologic differences between GD-related PTCs and PTCs identified in euthyroid patients, we investigated differences in prognostic histopathological parameters (histological features including BRAF-like or RAS-like tumor subgrouping, vascular invasion, lymphatic invasion, perineural invasion, extrathyroidal extension, lymph node metastasis, etc.) in N-GD and S-GD groups. Although there was a slight difference in select histopathological variables between the two groups (such as lymph node metastasis and lymphatic invasion), no single variable showed a statistically significant difference (p>0.05).

Another important finding is GD-related PTCs were enriched in subcentimeter low-risk tumors with a prevalence that varies depending on the presence of the underlying nodular thyroid tissue. Patients with N-GD had a higher risk of thyroid malignancy, consistent with the findings identified in some former reported series (6,8,18,19). Although not statistically significant, S-GD was enriched in BRAF-like tumors, whereas N-GD had an equal distribution with respect to RAS and BRAF-like tumors (43). Considering the higher malignancy risk for N-GD patients, surgical intervention may be a favorable option for the clinical management. This approach enables an evaluation of the potential malignancy and also aids in risk stratification. While most GD-related PTCs are associated with a low-risk disease, individual risk stratification is still indicated irrespective to the tumor size since subcentimeter papillary thyroid carcinomas are required to be subtyped as per the 2022 WHO classification of thyroid neoplasms (22) and other adverse features (e.g., angioinvasion, high-grade features) should be recorded.

## Conflict of Interest

The authors have no conflicts of interest to declare that are relevant to the content of this article

## Funding

The authors did not receive support from any organization for the submitted work.

## Ethics Approval

The study was approved by the Ondokuz Mayis University Faculty of Medicine, Medical Ethics Committee (IRB No: OMU KAEK 2019/431).

## Availability of Data and Material

All data is kept with corresponding author (MK).

## Code Availability

No custom code or software application was used during the preparation of the manuscript.

## Consent to Participate

Informed consent was not required due to the retrospective design of the study.

## Consent for Publication

All authors approved the final version and publication of the manuscript.
